# Emerging Neuroblastoma 3D *In Vitro* Models for Pre-Clinical Assessments

**DOI:** 10.3389/fimmu.2020.584214

**Published:** 2020-11-26

**Authors:** Diana Corallo, Stella Frabetti, Olivia Candini, Elisa Gregianin, Massimo Dominici, Horst Fischer, Sanja Aveic

**Affiliations:** ^1^ Neuroblastoma Laboratory, Istituto di Ricerca Pediatrica Fondazione Città della Speranza, Padova, Italy; ^2^ Rigenerand srl, Modena, Italy; ^3^ Division of Oncology, Department of Medical and Surgical Sciences for Children & Adults, University-Hospital of Modena and Reggio Emilia, Modena, Italy; ^4^ Department of Dental Materials and Biomaterials Research, RWTH Aachen University Hospital, Aachen, Germany

**Keywords:** 3D *in vitro* models, neuroblastoma, pediatric oncology, immunotherapy, drug screening, extracellular matrix

## Abstract

The potential of tumor three-dimensional (3D) *in vitro* models for the validation of existing or novel anti-cancer therapies has been largely recognized. During the last decade, diverse *in vitro* 3D cell systems have been proposed as a bridging link between two-dimensional (2D) cell cultures and *in vivo* animal models, both considered gold standards in pre-clinical settings. The latest awareness about the power of tailored therapies and cell-based therapies in eradicating tumor cells raises the need for versatile 3D cell culture systems through which we might rapidly understand the specificity of promising anti-cancer approaches. Yet, a faithful reproduction of the complex tumor microenvironment is demanding as it implies a suitable organization of several cell types and extracellular matrix components. The proposed 3D tumor models discussed here are expected to offer the required structural complexity while also assuring cost-effectiveness during pre-selection of the most promising therapies. As neuroblastoma is an extremely heterogenous extracranial solid tumor, translation from 2D cultures into innovative 3D *in vitro* systems is particularly challenging. In recent years, the number of 3D *in vitro* models mimicking native neuroblastoma tumors has been rapidly increasing. However, *in vitro* platforms that efficiently sustain patient-derived tumor cell growth, thus allowing comprehensive drug discovery studies on tailored therapies, are still lacking. In this review, the latest neuroblastoma 3D *in vitro* models are presented and their applicability for a more accurate prediction of therapy outcomes is discussed.

## Introduction

The turn of the 20th century was crucial for the development of the basic principles for *in vitro* cell growth enabled substantial biological discoveries. Over time, the complexity of *in vitro* systems has increased according to the needs of various branches of life science. The enhancement of *in vitro* techniques applicable in these two-dimensional (2D) systems greatly changed the perception of cell-related processes and allowed more accurate deciphering of the fundamental biomolecular and biophysical mechanisms active in both, physiological conditions and disease (1). The 21^st^ Century brought great progress in the development of the existing *in vitro* models for the study of more complex three-dimensional (3D) multicellular entities, while approaching as much as possible *in vivo* situations (2). A major tendency toward replacing, reducing, and refining (3R) animal use took place, supporting the application of the 3R principle for *in vivo* experimentation and energizing the development of diverse 3D cell culture technologies (3). This development represents the achievements of a breakthrough in the field of tissue engineering and regenerative biomedicine. Other disciplines of life science adopted the advances of available 3D models for addressing specific challenges and pitfalls encountered in the use of 2D systems, while also outlining novel considerations of cell and tissue-related questions.

In oncology, the introduction of 3D models for investigating tumor biology and cancer cells behavior is rapidly increasing. However, the standard procedures in this research still mainly follow a conventional route of initial testing in a Petri dish (2D) followed by *in vivo* validations in zebrafish, mice, or other small laboratory animals (4). The highly standardized protocols, well-established experimental approaches, and low costs of 2D tumor models explain the high rate of their application regardless of limited accuracy in representing native neoplastic tissues and predicting physiological values. The major obstacle to a straightforward translation of *in vitro* biological process analyzed in 2D conditions into an *in vivo* response is the lack of multicellular systems that are in direct contact with the cell-extracellular matrix (ECM) components (5). The tumor microenvironment (TME) is formed of several different cell types and non-cellular components (ECM). TME allows malignant cells to grow in 3D conditions, making the system extremely dynamic and complex. Yet, the proper architecture, tumor stiffness and relaxation behavior are not adequately considered in 2D *in vitro* studies, leading to limited information about the realistic changes in signaling pathways, metabolic activities, and genetic/epigenetic backgrounds of tumor and stromal cells (6, 7). The transition from 2D to *in vivo* pharmacological testing during the early stages of drug examination is therefore often critical but without the desired level of success (8). Many efforts are currently attempting to bridge the gap between 2D and *in vivo* systems by proposing different 3D *in vitro* models in which cell line and primary cells can be grown in either static or dynamic conditions.

Even though 3D cell culture techniques can minimize these limitations, their widespread use is still limited due to the relatively high costs, complexity of preparation, and lack of standardized protocols that can guarantee high reproducibility and unequivocal data interpretation (9). Unsurprisingly, 2D cell cultures and *in vivo* animal models are still considered the gold standards in pre-clinical settings in oncology. However, the traditional means of drug efficacy evaluation faces serious limitations, since many compounds that show good anti-cancer effects in murine models fail to provide meaningful clinical benefits for humans (10). Therefore, this scenario is changing in the direction of 3D models more often being built of primary cells as the protagonist of anti-neoplastic drug screening. This trend is also supported by important innovations in live cell *in vitro* imaging techniques that accelerate drug discovery (11). Still, most of these proposals come from cancers developing in adults, whereas there is a clear deficit of a pre-clinical 3D model providing analysis of drug response in pediatric tumors. This is particularly evident for neuroblastoma, for which a vast majority of scientific questions are still answered by using either 2D studies or the transgenic and xenograft zebrafish and murine models (12–14).

Neuroblastoma is an embryonal malignancy of the sympathetic nervous system with very heterogeneous biologic, morphologic, genetic, and clinical characteristics. It is classified as a neuroblastic tumor but in contrast to ganglioneuroblastoma and ganglioneuroma, neuroblastoma is more aggressive (15).

Based on several clinical and molecular risk factors each patient is stratified in one of the following risk groups: very-low, low, intermediate or high-risk (16). Such a pre-treatment risk group assignment facilitates treatment modalities as well (17). In high-risk patients, the aggressive course of the malignancy manifests as a disseminated disease (stage 4) with metastatic processes in the liver, bone marrow and bone, skin and several other organs (18). The treatment of these patients represents one of the most urgent challenges for oncologists. Despite intensive multimodal therapy, in more than 50% of high-risk patients, the disease progresses during the course of therapy leading to a fatal outcome (19).

Recent studies have shed light on the biology of neuroblastoma allowing a more accurate stratification of patients into risk groups, resulting in a reduction of treatment cytotoxicity without affecting the outcome of low and intermediate-risk patients (20, 21). However, the mortality rate of children in high-risk group is still high, and the development of more valuable therapeutic strategies remains urgent. During the last few years, different approaches such as transcriptomics analyses and genome-wide association studies have listed the genes associated with neuroblastoma susceptibility, aggressiveness, and progression (22, 23). The identification of such genes has raised the possibility of developing novel targeted therapies or reconsidering already existing drugs by the repositioning of FDA-approved drugs.

In this review, the latest *in vitro* 3D models suitable for assessing drug-specific responses in neuroblastoma will be addressed. We will discuss their implications in pre-clinical testing and applicability for a more accurate prediction of therapy outcomes. Finally, the possibilities of introducing already available bioengineered platforms and devices for the generation of predictive neuroblastoma models will be explored. We will assess current possibilities for a more accurate *in vitro* investigation of the pharmacotherapeutic cues in this tumor to justify clinical trials.

## Neuroblastoma *In Vitro* 3D Models

The lack of reliable *in vitro* tumor platforms for rapid and highly reproducible studies in cancer biology has driven the development of new tumor models by applying various bioengineering methodologies. Although these models share a common 3D conformation, each displays its own intrinsic property. In addition, the 3D models indicate unambiguously that the proliferation of tumor cells is significantly less when compared with 2D growth conditions (12, 24). In the following paragraphs, we will address the current applications of 3D *in vitro* culture systems in the neuroblastoma research field.

### Multicellular Tumor Spheroids (MCTSs)

MCTS is the most well-characterized 3D model for cancer research obtained by growing cancer cell lines under low adherent conditions (25). MCTSs can have different configurations depending on the specific aim of the study: they can be composed of a single or multiple cell types, generated either through the aggregation and compaction of multiple cells in suspension, or by establishing cell masses from a single cell *via* consecutive cell doublings. In either of these cases biomimetic ECM support, playing the role of a scaffold, may or may not be applied (12, 26).

MCTSs obtained by the aggregation of neuroblastoma cell lines represent an attractive tool to reproduce *in vitro* the *in vivo* characteristics of tumor cells with respect to the production of ECM, cell–cell interactions, growth kinetics, cellular heterogeneity, signal pathway activity, and gene expression (13, 25). Given the importance of the cell-ECM interaction in a 3D extent, among the most studied behaviors in the neuroblastoma field are the migratory and invasive potentials of cancer cells. For example, the analysis of different neuroblastoma cell lines embedded in 3D collagen gels revealed the relationship between cellular morphology (elongated/mesenchymal versus amoeboid/rounded cells) and their invasive capability through a surrounding environment (27). The main difference between the cells grown in 2D and 3D collagen structures is recognized in the Rac signaling pathway, which is differently expressed in these structures. It is a crucial regulator of cell invasion from the spheroid body through the surrounding matrix (27). These results highlight that biochemical signals in the neuroblastoma cells may change dramatically in response to changes in their spatio-temporal distribution. Moreover, they strengthen the case for using 3D systems to select the compounds able to counteract invasion of neuroblastoma cells. In addition to single chemical testing, neuroblastoma spheroids are also suitable for investigating the role of specific proteins on neuroblastoma outgrowth. For example, high levels of Stathmin (a Tubulin binding protein) are associated with tumor aggression and the appearance of metastatic disease. This protein has been selected by analyzing cell line-derived MCTSs where it contributes to a higher invasive motility of the cells (28). Besides Stathmin, SNAI2 is also a crucial molecular determinant of invasive tumor strands. This protein defines the border regularity of the MCTSs and promotes local 3D invasion and dissemination of neuroblastoma cells (29).

However, some critical issues related to cell line-derived MCTSs need to be considered. The variability in spheroid size and their inhomogeneous density profoundly affect the response to drug treatments. As a consequence, this feature negatively impacts the reproducibility and reliability of the obtained results (26). In addition, long-term *in vitro* culture of cell line-derived MCTSs is very challenging since these structures lack a stem cell population able to self-renew the spheroid necrotic core. Moreover, this model is not able to faithfully approximate/simulate the complexity of neuroblastoma genetics and the tumor microenvironment found in humans.

### Tissue-Derived Tumor Spheres (TDTSs)

TDTSs are obtained by tumor tissue mechanical dissociation (26). Due to the origin of primary cells, this *in vitro* model system more closely reflects the genetic and clonal heterogeneity of the native tumor, thus providing a more accurate pre-clinical platform (30). Despite the fact that these tailored models can lead to an improved level of prediction, their development and application are still a challenge as sample collection and size, protocol standardization and data reproducibility are critical issues for neuroblastoma. More easily established are neuroblastoma-derived spheroids generated from the bone marrow aspirates of patients diagnosed with stage 4 metastatic disease (30). In some cases, surgically removed tumor resections also allow *in vitro* reproduction of neuroblastoma. However, at the moment it is hard to predict which clinico-biological criterion is a determining factor in enabling the successful *in vitro* growth of a single specimen (31). In fact, a success rate of 55% has been reported for neurosphere maintenance *in vitro.* Also, the expansion of neurospheres cannot be linearly predicted from patient clinic data such as age, stage, *MYCN* amplification and the presence of segmental chromosomal aberrations (31). While the characterization of the resulting neurospheres is currently limited to the expression of CD56 and GD2 neuroblastoma markers, their importance relies on the presentation of reproducible protocols for the *in vitro* expansion of often limited amounts of tumor tissue specimens. Screening for additional antigens specific for neuroblastoma is, however, necessary for more accurate selection of tumor cells with stem features that are often responsible for drug resistance development and disease recurrence. Another TDTS model resembling neuroblastoma intratumoral heterogeneity has been reported by Thole and colleagues (30). The primary neuroblastoma TDTSs culture derived from a bone marrow aspirate with 80% tumor cell infiltration can be cultured in Matrigel. Neuroblastoma cells grown as 3D spheroids maintain the tumorigenic capability in a xenotransplantation mouse model through five passages. Importantly, these TDTSs model systems partly reflect the genetic and clonal heterogeneity of the initial biopsy (30). Altogether, the reported neuroblastoma TDTSs represent essential initial steps toward more sophisticated 3D neuroblastoma modeling suitable for pre-clinical testing.

### Patient-Derived Tumor Organoids (PDTOs)

Organoids are *in vitro* derived 3D cell aggregates that are capable of self-renewal and self-organization, while exhibiting expected organ functionality. Organoids are usually generated from either embryonic stem cells (ECS) or induced pluripotent stem cells (iPSC) (32). To date, several organoids have been established for many cancer types (reviewed in ref. (33)).

PDTOs cultures show strong phenotypical and genetic similarities to the original tumor, enabling their use across a wide spectrum of applications. PDTOs allow long-term culture and cryopreservation for the generation of patient-derived tumor organoid biobanks (34). However, most of the patient-derived cancer organoids have an epithelial origin. The generation of organoid cultures from primary neuroblastoma samples, as well from other non-epithelial cancers, remains today a major challenge in organoid technology.

## Moving from 2D Toward 3D Pre-Clinical Settings

**Figure 1:**
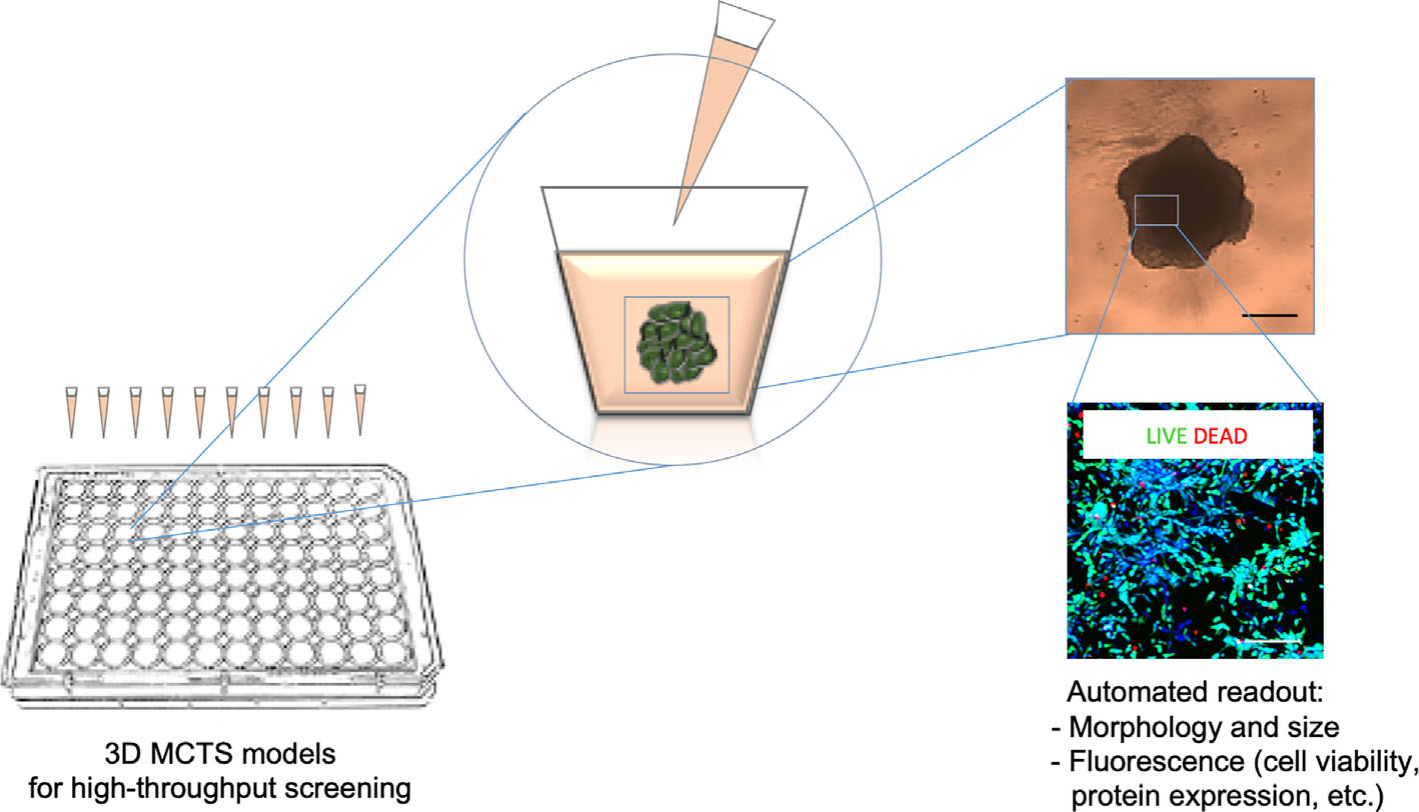
Scheme of the HTS (high throughput drug screening) method. HTS is used to evaluate multiple morphological and cellular parameters in a high number of MCTSs (multicellular tumor spheroids) grown and treated inside microplates. The drug treatment is combined with an optical (upper image) and fluorescence (lower image) microscopy systems for automated image acquisition and coupled analyses through specific software pipelines. These platforms make suitable automatic quantitative analyses of the 3D culture systems in response to drug administration.

In general, we are currently faced with an extremely low efficacy of pre-clinical studies. In oncology, less than 10% of drugs successfully conclude clinical trials (35), resulting in significant time and economic loss. The introduction of high-throughput drug screening (HTS) speeds up target identification and lead compound selection, increasing the number of anti-neoplastic compounds that potentially reach clinical trial (36). As stated above, the majority of HTS studies are based on the use of tumor cell lines grown in 2D conditions. This approach is slowly being reconsidered and comprises the introduction of different 3D cultures in order to increase the likelihood of pre-clinical success (**Figure 1**). In neuroblastoma, the number of studies that have examined 3D spheroids for HTS is relatively low and their introduction is a challenge. This is particularly true for 3D structures containing patient-derived primary cells due to a lack of study material and difficulties to culture and maintain them *in vitro*.

Each tumor can be considered as a heterogeneous structure deriving from the interaction between cancer cells and the surrounding microenvironment, which provides important physical and biochemical signals for its growth. The information coming from the native 3D human cancer structure can affect, for example, the expression of specific genes as well as the diffusion of nutrients and oxygen within the tumor mass. Moreover, this complex network of interactions determines the ability of tumor cells to resist and escape from pharmacological treatments. This diversity could be at the root of treatment failures, which remain a peril in the neuroblastoma field today.

### Pioneering 3D Neuroblastoma Models for Drug Screening Studies

In order to address these needs, 3D culture technology has been applied in the testing of the sensitivity of several MCTSs to doxorubicin exposure, confirming the modification in tolerance to this drug when moving from 2D to 3D culture systems (37). Moreover, MCTSs obtained with the SH-SY5Y neuroblastoma cell line shows higher sensitivity to rapamycin and triciribine when compared to the correspondent 2D culture, emphasizing the importance of *in vitro* 3D models as a valid system for initial testing of new anti-cancer agents (38). In a recent work aimed at identifying candidate drugs for repositioning in high-risk patients, HTS of a library of anticancer compounds was tested in neuroblastoma MCTSs (39). This study proposes MCTSs viability validation using a high-content imaging approach as a powerful and reliable 3D platform to predict pre-clinical efficacies and to reproduce drug responses of neuroblastoma tumors.

## Biomimetic Extracellular Matrix (ECM) Supports for Neuroblastoma Tumor Model Fabrication

The ECM represents a dynamic and versatile network of secreted proteins and polysaccharides assembled together in an organized network and having distinct roles in cell biology and tissue homeostasis (40). The ECM provides structural support to all organs and provides a substrate upon which cells can migrate. Moreover, the interaction between cells and the ECM macromolecules plays an essential role in tuning the behavior of many cell types in a physiological context. Indeed, during embryonic development the ECM provides essential extrinsic signals for the correct migration of neural crest cells, the pluripotent stem cell population from which neuroblastoma may arise (41).

### Role of ECM in Neuroblastoma

The ECM has a complex and tissue-specific molecular composition. The dynamic remodeling of the ECM is of outmost importance in order to determine the specificity of its biological functions during organogenesis and to guarantee a proper tissue homeostasis. As a consequence, the disruption of such mechanisms disorganizes the extracellular niche, leading to abnormal behaviors of resident cells and the failure of tissue homeostasis. Indeed, dysregulation of ECM composition, architecture and stiffness leads toward development or worsening of several diseases, including fibrosis and cancer (42). A large body of experimental evidence emphasizes how ECM proteins promote tumor metastasis and modulate the maintenance and expansion of several cancer cell types and metastatic niches (reviewed in ref. (43–45)).

In neuroblastoma, the presence of a stromal component positively correlates with tumor maturation and favorable prognosis (46). In addition, the deposition of specific ECM components defines an ultra-high risk group of patients affected by neuroblastoma, suggesting that the quantification of tumor stroma components by morphometric techniques could be a valuable tool in improving patients’ risk stratification (47). From the molecular perspective, several studies have demonstrated how the cross-talk between neuroblastoma cells and the ECM influences cancer cell differentiation (48). More recently, besides the molecular signaling activated through the cell-ECM interaction, the biomechanical properties of the ECM, such as stiffness and deformability, have also been recognized as mechanical modulators of cancer cell behavior (49). Indeed, dissecting the role of the ECM within the neuroblastoma niche may provide insight into new mechanobiological cues influencing tumor growth and differentiation. This knowledge would provide the basis for future work aimed at the design and exploitation of novel therapeutic strategies against neuroblastoma.

### Cast *In Vitro* 3D Models for Studying Neuroblastoma

**Figure 2:**
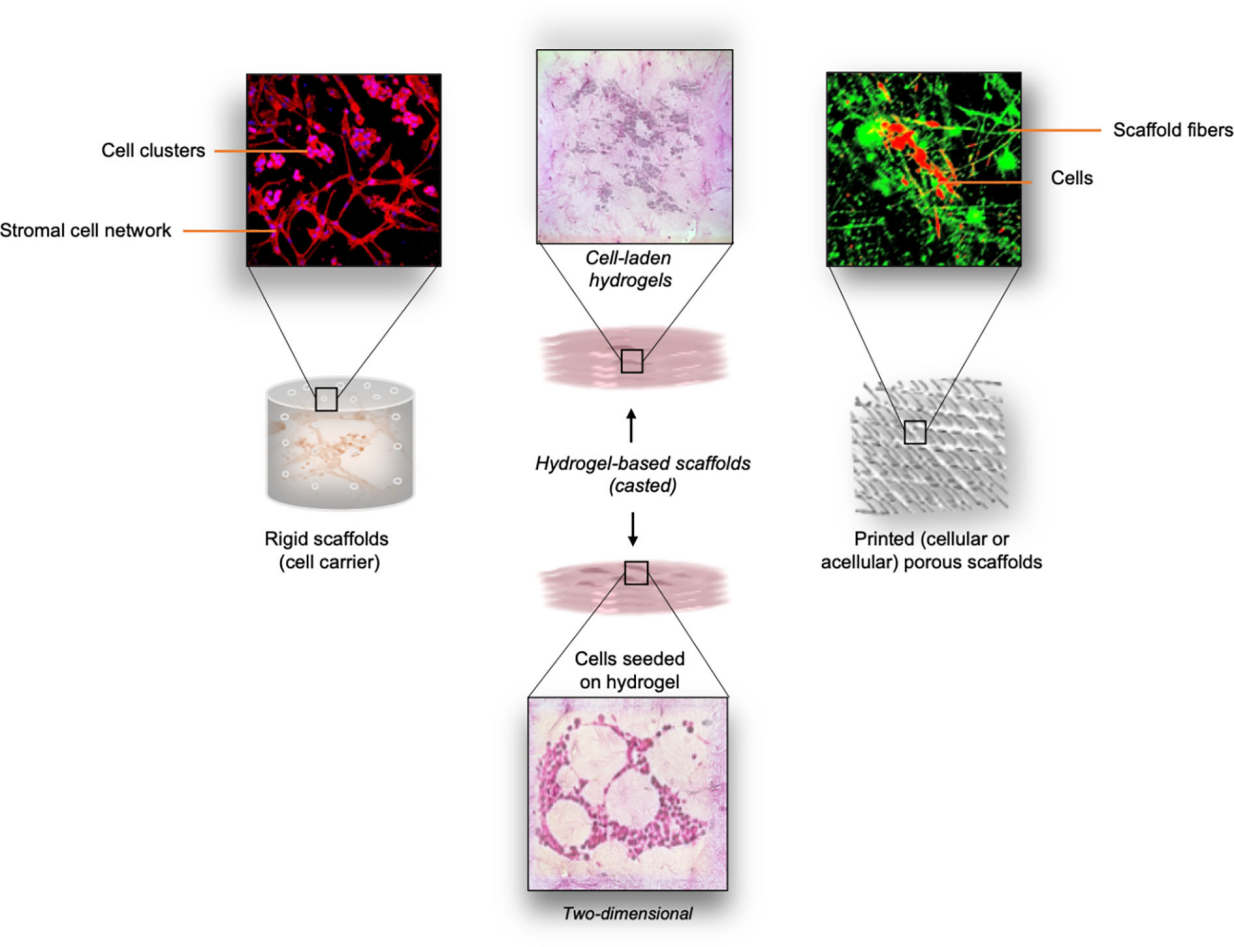
Overview of different types of scaffolds explored for neuroblastoma studies. *Left panel:* Thermal sintering-based approach used for the fabrication of cell-free (rigid) scaffolds with defined geometry. These scaffolds provide mechanical support for cell growth. Cell morphology and cell distribution inside the interconnecting microchannels is directly influenced by the structure of the scaffold. *Middle panel:* Cast cell-laden hydrogels are used as the biomimetic ECM support for the embedded cells. As an option, cells can be seeded on top of the pre-made hydrogel structure*. Right panel:* Printing (e.g. microextrusion, drop-on-demand, laser-based printing) of various bioinks can be adopted for the scaffolding process. Both cellular and acellular approaches can be adopted for the generation of porous scaffolds with defined spatial distribution of the bioink.

As mentioned, after important tumor-related knowledge was obtained from 2D cell systems, the importance of introducing ECM component as important determinant of tumor cells behavior pushed the boundaries beyond the second dimension (50). This led to the incorporation of the achievements obtained in the bioengineering field, where different biomimetic matrices have been developed. The full range of available materials, natural, synthetic and semisynthetic (hybrid), have been exploited in the form of hydrogels for their characteristics as a suitable ECM support for tumor cell growth and their autonomous self-assembly in tissue-like structures (**Figure 2**).

The detailed classification of the hydrogels, their applicability and suitable processing approaches have been summarized by Ullah and colleagues (51, 52). The main demands these materials must satisfy are: i) provide proper cell alignment and attachment, ii) sustain cellular metabolic activities, and iii) mimic cell response to mechanical and chemical stimuli as in tissue (53). The cells can be either seeded on the porous scaffold that provides them with 3D support or encapsulated directly within the biomaterials (cell-laden hydrogels) with well-defined stiffness and viscosity. The most commonly used biomaterials for the production of the scaffolds or cell-laden constructs are: collagen, hyaluronic acid (HA), alginate, agarose, gelatin, fibrinogen (natural); poly(lactic-co-glycolide) (PLGA), polyethylene glycol (PEG), poloxamer 407 (Pluronic F127), and polycaprolactone (PCL) (synthetic); and methacrylated gelatin (GelMA) (semisynthetic) (54, 55). The choice is determined by the tumor type and also by specific physical parameters such as elasticity and stiffness (56). In this context, the ways in which biomimetic matrices with different mechanical and biochemical cues can determine the neuroblastoma cell phenotype have been investigated, along with their contribution to the spatio-temporal tumor cell organization or response to drugs. The excellent reproducibility of the *in vivo* data has been demonstrated for the neuroblastoma cell lines Kelly and their cisplatin resilient counterpart (Cis83) when grown on different chemical modifications of collagen, one with glycosaminoglycan (Collagen-GAG) and the other with nanohydroxyapatite (Collagen-nHA) (57). When treated with cisplatin, the cells grown in 3D conditions show similarities with the PDX (patient derived xenograft) treatment, while differing substantially from their 2D control. This finding strengthens the use of 3D models for initial drug evaluations since they more closely approximate the expected response *in vivo.* Bacterial nanocellulose scaffolds coated with collagen is another approach that potentiates SH-SY5Y adhesion in 3D geometric conditions (58). Physical support for neuroblastoma cell growth is also provided by electrospun fibers used as 3D matrices (59). Micro- and nano-fibers created by electrospinning guarantee high porosity of the structures and favor neuroblastoma cell proliferation and adhesion, while promoting neurite out-growth (60). The usefulness of the highly aligned graphene-augmented inorganic nanofiber (GAIN) scaffolds for biomedical cancer research has also been proven for several tumor cell types including neuroblastoma (61). Although they do not allow tumor-like 3D cell organization entirely, these scaffolds open an opportunity for a fast and highly reproducible validation of anti-cancer drugs oriented toward the modulation of cell migration. Another application of graphene is in the fabrication of nanocomposite hydrogel scaffolds in which the magnetic nanoparticle-decorated reduced-graphene oxide (m-rGO) nanosheets lead to a unidirectional orientation of the cells (62). This approach is particularly interesting in the models where both cell orientation and the conductivity of the biomaterials are required (63). On the other hand, the possibility of growing neuroblastoma cells in collagen-based hydrogels opens another prospect for achieving the 3D structures of neuroblastoma for pre-clinical examinations. Collagen-based structures also allow the reproduction of a 3D microenvironment suitable for better comprehension of pro-migratory pathways activated in neuroblastoma cells (27). Moreover, collagen-based scaffolds offer possibilities for examination of the efficacy of a new class of drugs known as migrastatics (64).

Neuroblastoma cells with different molecular backgrounds show distinct patterns of growth inside biomimetic 3D structures. Moreover, neuroblastoma cells can also be cultured in a mixture of collagen and agarose that is often proposed in order to modify the mechanical properties of pure collagen (65). The encapsulation of neuroblastoma cells is as well supported by alginate and gelatin (66). Either of the cast 3D platforms mentioned can be downscaled thus opening the possibility of HTS applications. In fact, collagen microencapsulation is a highly controllable approach for obtaining miniaturized neuroblastoma tumors (67). In this nanofibrous collagen meshwork, a reconstruction of the neuroblastoma microenvironment is achieved thanks to the stromal cells’ support. This study opens the possibility for using neuroblastoma cell-laden bioinks for the reproduction of miniaturized tumors applying different printing methodologies.

### (Bio)Printing Neuroblastoma 3D Models

**Figure 3:**
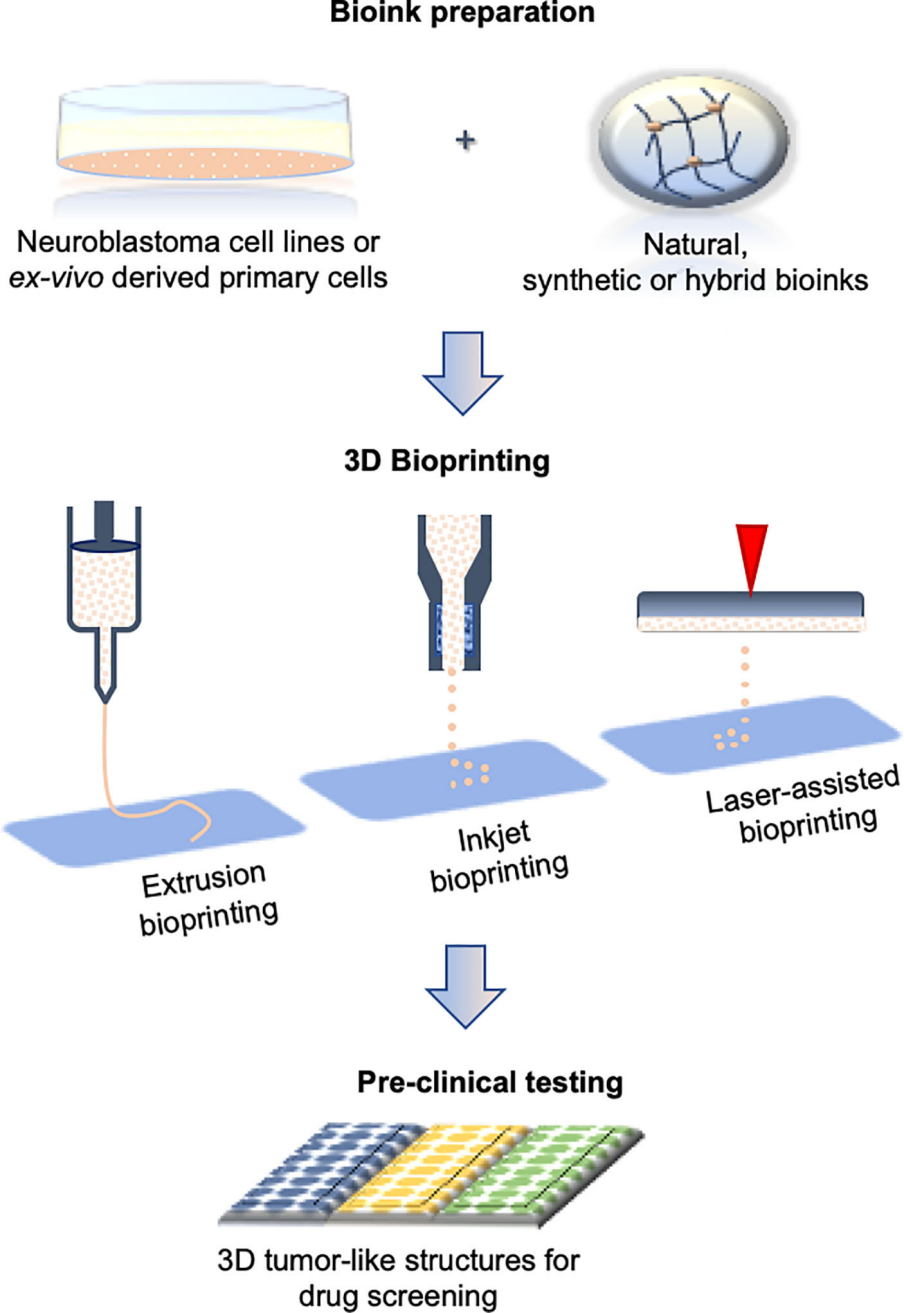
Bioprinting process during *in vitro* 3D model generation. The steps include bioink preparation (cells embedded in biocompatible inks), 3D bioprinting (three most commonly used printing techniques are presented), and drug screening. All the steps can be performed in automated manner.

Various 3D printing techniques have been employed to develop more accurate constructs for cell-growth supports (68). Printing technology is challenging the faithful reproduction of the tissue compartments at the microscale while maintaining their unique spatio-temporal organization (69). A parallel expansion of the array of printable biomaterials compatible for research activities or medical requirements has broadened the possibilities for 3D printing (70). The inclusion of (bio)printing methodologies in the neuroblastoma field has been considered in a few studies thus far (**Figure 3**).

A mix of GelMA and methacrylated alginate (AlgMA) have allowed the optimal mechanical properties and porosity for the growth of neuroblastoma cells (71). Similar to the collagen-based hydrogels, this printable bioink also permits tumor cells to organize and create 3D architectures that very closely mimic human neuroblastoma. An optimal level of stability upon printing is also possible with chitosan-gelatin ink, which shows good biocompatibility and allows the proper adhesion of neuroblastoma cells. It is also easily manageable without the need for additional processing post-printing, which makes it a good candidate for high rate production of cell-laden hydrogels (72).

Different neuroblastoma cell lines have been explored using the freeform reversible embedding of suspended hydrogel (FRESH) bioprinting method for conductive scaffolds (73). Although the study was designed for neurodegenerative diseases, the approach and experimental design are incredibly attractive for analyzing tumor cells within 3D conductive bioinks. The FRESH method proves that high resolution of neuroblastoma 3D structures can be obtained using low-viscosity liquids in a supporting bath of gelatin. Neuroblastoma cells can be successfully grown in cellulose and alginate-based hydrogels, demonstrating the applicability of FRESH bioprinting for the generation of microsized 3D neuroblastoma tumors (74). The same type of hydrogel has been explored in the immunology field as well showing the changes in immunophenotype profiles in neuroblastoma cells surrounded by biomimetic ECM (75). Beta tricalcium phosphate (β-TCP) scaffolds used as bone mimetic, have been obtained by combining high resolution 3D printing with manually cast slurry (76). They have been proposed as a suitable approach for sustaining neuroblastoma cell growth inside the metastatic niche (24). In this manner, a local microenvironment is guaranteed allowing the quiescence of tumor stem cells. In this model, the stromal support has been confirmed as a substantial factor in tumor cell organization along with the geometry of the scaffolds. Although the model has not yet been exploited for pre-clinical studies, it can be easily adapted for low/medium scale of drug screening. The metabolic activities and cell death ratio can be easily measured, while microscopy would require special adaptation in the case of live-imaging acquisition.

## Engineered Platforms for Studying Tumor Biology, Immunology and Drug Efficacy

The aim of engineered disease models is to reproduce *in vitro* the complexity of the pathological environment in order to gain a better understanding of disease etiology and progression (77). The model composition depends on the research objectives: the more complex the phenomenon under investigation, the more elaborate the model must be (78). *In vitro* cell modeling using miniaturized bioreactors shows great advantages since they allow the use of small volumes of reagents and low cell number, the portability, design versatility and integration with existing devices or platforms for HTS (79). To study the effects of a static magnetic field on SH-SY5Y neuroblastoma cells, a miniaturized optically accessible bioreactor (MOAB) has been developed based on a prototype of Raimondi et al. (80). This bioreactor is composed of three independent and magnetically lockable culture chambers, each containing a polystyrene scaffold assembled on the top surface of a main body structure. The MOAB is provided with two magnets, located in the chambers and in the main body, whose magnetic coupling ensures a hydraulic seal during the perfusion of 3D constructs. This magnetic seal generates a static magnetic field, which influences cell functions including viability, metabolic activity and gene expression. The MOAB device, specifically conceived to study the influence of a static magnetic field on neuroblastoma cell lines, might potentially be used as an *in vitro* model of neurodegeneration to test perfused 3D cell constructs in terms of response to different stimuli.

To reproduce neuroblastoma vasculogenic mimicry, a complex *in vitro* model has been fabricated by culturing pre-vascularized neuroblastoma cell sheets separated by fibrin layers in a perfusion bioreactor. The cell sheets are prepared by co-culturing the neuroblastoma cell line SK-N-BE(2) with human umbilical vein endothelial cells (HUVEC) on temperature-responsive poly(N-isopropylacrylamide) (PIPAAm)-grafted culture plates. A collagen-gel base with microchannels is used as a support for the vascular bed (81). This platform may represent an interesting option for drug testing, especially for drugs exhibiting antiangiogenic features. The fabrication of an intrinsic system of vasculature allows a better mimicking of the native tumor while augmenting predictive power for translation into pre-clinical applications. However, the difficulty of cell-sheet fabrication and stacking, together with the assembly of the collagen-gel base with microchannels for the perfusion, represent a critical obstacle to a large-scale application of this model.

Another step toward a model resembling native neuroblastomas is represented by the 3D tetra-culture brain microphysiological system (BMPS) used to test neurotoxic chemical agents. This system is developed starting from the OrganoPlate (MIMETAS, Netherlands) in which neuroblastoma cells (N2a), astrocytes (C8-D1A) and microglia (BV-2) are cultured in a collagen type I solution to recreate the brain parenchyma. The neurovascular environment is assured by also including endothelial cells (bEnd.3). The entire system requires culturing in perfusion conditions to permit appropriate 3D cell organization. This plate-based microfluidic platform may be applied for automated, high-throughput and high-content imaging with relatively fast readouts (82). Nevertheless, more consistent validations of their use for a routine drug screening are mandatory.

### Clinical Needs and Future Perspectives for *In Vitro* Immunotherapy Evaluations

Immunotherapies have recently attracted great interest as a novel approach for cancer treatment, but the lack of adequate *in vitro* models for testing the efficacy of these therapies at a personalized level is still an issue (75, 83). Immunotherapy strategies rely both, on the ability of the immune system to kill malignant cells by recognizing specific tumor antigens and the ability of tumor cells to evade this physiological defense system. Two major strategies can be identified: re-activation of the tumor infiltrating lymphocytes (TILs) through the immune checkpoint inhibitors and chimeric antigen receptor-T (CAR-T) or T cell receptor (TCR) cell therapy.

Checkpoint inhibitors work by blocking the inhibitory binding between T cells’ checkpoint proteins (e.g. PD1) and their ligand on tumor cells (e.g. PDL1), allowing the immune system to become able once again to kill cancer (84). The CAR-T/TCR consists of the patient’s T-cells genetically modified to express unique tumor antigens that give them the ability to specifically target cancer cells, such as GD2 for neuroblastoma (84, 85).

**Figure 4:**
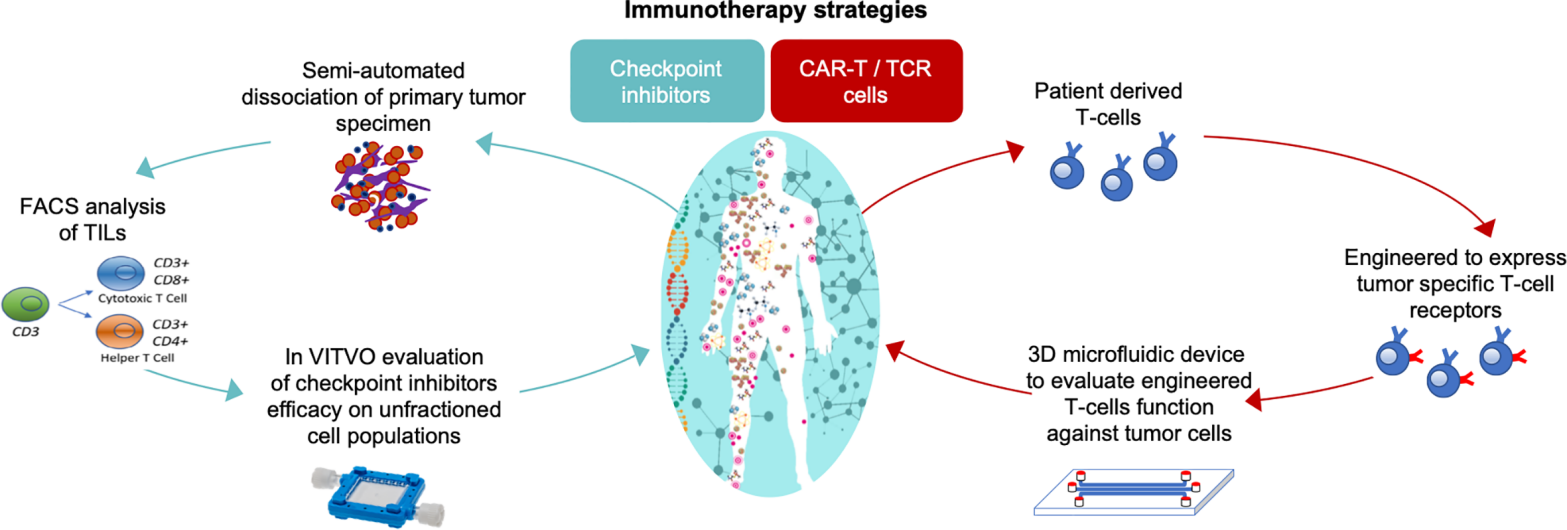
*In vitro* 3D models to test the efficacy of immunotherapeutics in a personalized approach strategy. On the left, the functional assay for cancer responsiveness to checkpoint inhibitors using VITVO bioreactor described by Candini et al. (88); on the right, TCR T-cell activity against tumor using 3D microfluidic device described by Pavesi and colleagues (83).

Currently, several types of immunotherapy are being studied for use against neuroblastoma (86, 87) and complex *in vitro* 3D models that will allow a close relationship between target and effectors as occurs *in vivo* are needed for pre-clinical efficacy evaluation of immunotherapeutics. As shown in **Figure 4**, immunotherapy strategies can be developed and tested directly using patient derived cells as part of a personalized medicine approach.

To test the migratory and lethality of TCR engineered patient-derived T cells toward hepatic tumors, an interesting 3D *in vitro* model has been developed by Pavesi and colleagues (83). This model consists of a 3D microdevice made of a poly(dimethylsiloxane) structure comprising a gel region with media channels separated from the gel channel by trapezoidal posts. Tumor cells are cultured embedded in a type I collagen gel solution that is injected into the predefined gel region of the device. The culture medium channels allow the cell culture perfusion and the free movement of TCR-T cells from the medium channel into the 3D solid collagen region containing target cells. This 3D assay could lead to a better understanding of what is encountered physiologically during adoptive T cell therapy of solid tumors, where the chemotactic characteristics and intrinsic killing of the engineered T cells are key factors in the successful outcome of the therapy. Although this 3D microdevice has been tested with human liver carcinoma cell line as its target, it could be useful to study other solid tumors including neuroblastoma.

For functional *in vitro* prediction of the efficacy of checkpoint inhibitors, a rapid functional test based on the use of the VITVO device (Rigenerand srl, Italy) has been recently proposed. VITVO is a small, portable bioreactor integrating a synthetic and biocompatible fiber-based matrix, and can host several types of cells, also in combination. Using this platform, primary cells harvested from human lung cancer specimens have been evaluated to predict the patient specific anti-tumor immunity of TILs triggered by checkpoint inhibitor Nivolumab (88). The same approach could also be considered to evaluate neuroblastoma responsiveness to immunomodulatory agents. Although these *in vitro* 3D platforms potentially offer innovative tools for the development of fast and reliable personalized assays, further studies are needed to confirm their relevance for clinical use.

## Conclusions

**Figure 5:**
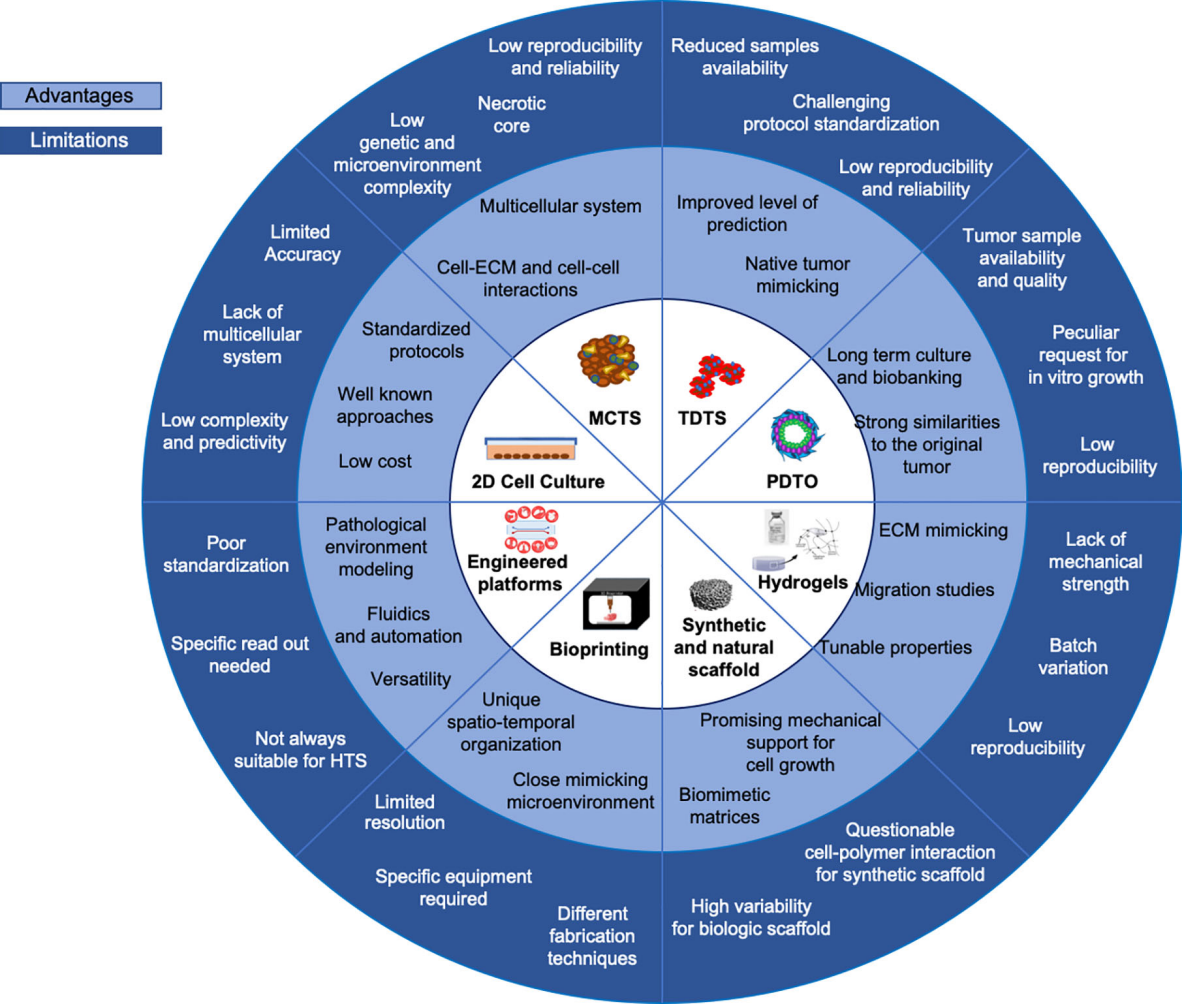
Current *in vitro* models. Advantages and limitations overview of current 3D *in vitro* models versus 2D systems are summarized.

The more effective targeting of malignant cells remains a highly challenging task since the existing therapeutics approaches do not adequately achieve the same efficacy of *in vitro* determined efficacy when translated to clinical trial. The purpose of including 3D tumor models is therefore to establish a new approach that overcomes the limitations of currently used *in vitro* protocols (**Figure 5**). The 3D *in vitro* models provide a closer reflection of the complexity of malignant tissues by nurturing complex cell-cell and cell-ECM organization. Under these growth modalities, tumor cells more closely approach their native interactions, generating tumor-like structures that strongly define the types of response to toxic drug insults. The number of possibilities for using neuroblastoma 3D models in HTS is increasing thanks to advances in bioengineering field. However, the automation of multiparametric data extrapolation in terms of volumetric parameters and cell viability within analyzed 3D structures remains a challenge. The introduction of bioprinting processes in pre-clinical studies is expected to bring to a greater reproducibility of the cell models, as well as higher predictability and controllability of the structures in comparison with the cast approach. The precision and resolution of the cell-laden structure bioprinting are determined by the characteristics of the nozzles, which do not allow the printability of all currently available bioinks. Although very useful, a limitation of using preformed porous scaffolds for sequential cell plating and culturing is the poor reproducibility of the spatio-temporally location of more than one cell type. The combination of (bio)printing and fabrication of microfluidic platforms in the field of neuroblastoma can therefore amplify the possibilities for HTS in 3D conditions. It is particularly intriguing if the described, versatile 3D cell culture systems could advance the pre-clinical evaluation of newly proposed tailored therapies and cell-based therapies that are currently under investigation for defeating neuroblastoma tumor cells.

## Author Contributions

DC, SA, OC, and SF wrote the manuscript. DC, SF, SA, and OC reviewed the literature and collected available data. CD, SA, OC, EG, and SF designed the figures. HF and MD revised the manuscript. All authors contributed to the article and approved the submitted version.

## Funding

This work was supported by the Fondazione Italiana per la Lotta al Neuroblastoma (project number 19_20FNBL).

## Conflict of Interest

MD is founder and member of the Board of Directors of Rigenerand srl, a University start-up company. MD interests are managed by the University of Modena and Reggio Emilia in accordance with their conflict of interest policies. SF, OC, and EG are currently employed by Rigenerand srl.

The remaining authors declare that the research was conducted in the absence of any commercial or financial relationships that could be construed as a potential conflict of interest.
